# Assessing the multidimensional burden of facioscapulohumeral muscular dystrophy through patient-reported outcomes and experience

**DOI:** 10.1186/s41687-026-01026-z

**Published:** 2026-02-25

**Authors:** Wenjing Ji, Yifei Zhao, Yuqiang Kang, Zikai He, Linkai Xu, Sundus Shukar, Ailiyaer Aikepaier, Xinyue Chen, Qifang He, Minghui Zeng, Yuhua Lin, Minting Lin, Huxinigeer Maimaiti, Tingting Liu, Linguo Li, Zhiqiang Wang, Yu Fang

**Affiliations:** 1 Center for Drug Safety and Policy Research, Xi’an Jiaotong University, Shaanxi, China; 2 Department of Pharmacy Administration, School of Pharmacy, Xi’an Jiaotong University, Shaanxi, China; 3 School of Pharmacy, Xi’an Jiaotong University, Shaanxi, China; 4 Chinese Organization for Rare Disorders, Hangzhou, Zhejiang China; 5 Department of Neurology and Institute of Neurology of First Affiliated Hospital, Institute of Neuroscience, and Fujian Key Laboratory of Molecular Neurology, Fujian Medical University, Fuzhou, 350005 China; 6 National Regional Medical Center, Department of Neurology, Binhai Campus of the First Affiliated Hospital, Fujian Medical University, Fuzhou, 350212 China

**Keywords:** Facioscapulohumeral muscular dystrophy, Patient-reported outcomes, Disease burden, Qualitative research, Quantitative study

## Abstract

**Background:**

Facioscapulohumeral muscular dystrophy (FSHD) is a rare, autosomal dominant disorder that adversely affects life expectancy and health-related quality of life. There is a paucity of comprehensive research on FSHD, particularly within the Chinese population. This study aims to explore how FSHD affects this population.

**Methods:**

The study employed a convergent parallel mixed-methods design, consisting of a quantitative study and a qualitative study. The quantitative phase involved a cross-sectional online survey conducted between March and October 2024. The self-administered questionnaire includes demographic characteristics, patient diagnosis process, disease progression, treatment, quality of life, mental health, and unmet needs. For the qualitative study, four researchers conducted thirty-three semi-structured, in-depth online interviews with FSHD patients. Patients with clinically and genetically confirmed FSHD were recruited nationwide through the FSHD Community Network—a patient-led advocacy organization affiliated with the Chinese Organization for Rare Disorders (CORD). The study is registered at ClinicalTrials.gov (identifier: NCT06517498). Interviews were audio-recorded, transcribed, and analyzed using thematic analysis with NVivo software.

**Results:**

This study examined the physical, psychological, and economic burden, as well as the quality of life, of patients with FSHD. Diagnostic delay was common, with an average diagnostic timeline of 9.8 years and a misdiagnosis rate of 57%. Significant familial clustering posed considerable challenges, with as many as 23 patients reporting over five family members exhibiting FSHD-related symptoms. More than 70% of patients indicated that the disease moderately to severely impacted their lives, ranging from sleep to daily activities. Moderate-to-severe anxiety and depression were reported by nearly half and 69.6% of participants, respectively. The mean EQ-5D-5L utility index was 0.542, and the median Visual Analog Scale score was 50. Participants prioritized the following unmet needs: approval of FSHD-targeted medications, access to assistive devices, psychological support, employment opportunities, and a more accommodating social environment.

**Conclusions:**

The findings highlight a substantial disease burden and significant unmet needs among FSHD patients. Early diagnosis, approval of targeted therapies, and comprehensive support from families, healthcare systems, and society are imperative. These measures are essential to improve health outcomes and quality of life for this patient population.

**Supplementary Information:**

The online version contains supplementary material available at 10.1186/s41687-026-01026-z.

## Introduction

Facioscapulohumeral muscular dystrophy (FSHD) is an inherited myopathic disorder characterized by progressive, asymmetric weakness and atrophy predominantly affecting facial, scapular stabilizer, humeral, truncal, and lower-extremity musculature [1]. FSHD is the third most common type of adult-onset muscular dystrophy, behind Duchenne and Becker muscular dystrophies and myotonic dystrophy [2]. The estimated prevalence of FSHD is about 4 to 12 cases per 100,000 individuals [3, 4], while in China, it is 0.75 per million from 2001 to 2020, and it increased from 0.22 in 2001–2015 to 0.53 per million in 2016–2020 [5]. The disorder demonstrates marked interindividual and intramuscular heterogeneity, with variable spatial progression patterns both among patients and within individual musculature [1]. Crucially, muscular deterioration follows a characteristically progressive trajectory throughout the disease course.

FSHD occurs due to epigenetic de-repression of the *DUX4* gene, which leads to aberrant expression of the transcription factor DUX4 resulting in weakness of skeletal muscles [6, 7]. Recent advances in elucidating the molecular pathophysiology of FSHD have catalyzed the development of targeted therapeutic strategies directed at suppressing *DUX4* expression, with several agents progressing through preclinical and clinical trial phases [8]. Validation of clinically relevant endpoints—including robust functional outcome measures, quantifiable biomarkers, and objective progression metrics (e.g., magnetic resonance imaging [MRI] and ultrasonography)—constitutes a critical prerequisite for therapeutic development [9]. Notwithstanding its investigational promise, losmapimod (a p38α/β mitogen-activated protein kinase inhibitor) failed to meet its primary efficacy endpoint versus placebo at week 48 in the Phase 3 REACH clinical trial [10]. Alternative therapeutic approaches, including an investigational RNA-targeted therapeutic by Avidity Biosciences, are currently undergoing evaluation, with multiple novel modalities under active development [11]. Present clinical management remains exclusively supportive, focusing on preservation of functional capacity and optimization of quality of life [8].

Patients with rare diseases, such as FSHD, face a arange of complex clinical and systemic challenges. These include diagnostic delays, a lack of targeted therapies, barriers in insurance coverage, and considerable psychological morbidity [12]. Significant evidence gaps remain concerning patient-reported outcomes (PROs) specific to FSHD. PROs are defined as any report of a patient’s health status that comes directly from the patient, without interpretation by clinicians [13]. Patient-Reported Measures (PROMs) are the instruments used to measure PROs, such as health-related quality of life (HRQoL). Collecting robust PRO data is essential for healthcare systems and therapeutic developers to [1] quantify disease burden [2], optimize care models, and [3] implement evidence-based coping interventions. Therefore, this study aimed to explore patients’ perspectives and experiences regarding the impact of FSHD on their lives, as well as their unmet needs across diagnostic and treatment pathways, physical and mental health, and social support and integration.

## Methods

### Study design

The study utilized a convergent parallel mixed methods design, comprising: (a) a quantitative study; (b) a qualitative phase. Both components were conducted during an overlapping time-period and analyzed independently. The findings were integrated at the interpretation phase to provide a comprehensive understanding of patients’ perspectives and lived experiences regarding FSHD.

### Participant recruitment

Patients with clinically and genetically confirmed FSHD were recruited nationwide for both the qualitative and quantitative phases of the study through the FSHD Community Network—a patient-led advocacy organization affiliated with the Chinese Organization for Rare Disorders (CORD). Prior to enrollment, all participants received comprehensive study information and provided informed consent. Communications were conducted via WeChat (Tencent Holdings Ltd., Shenzhen, China), the dominant instant messaging platform in China, using text messages or voice calls. Individuals who did not respond after three contact attempts within one week were excluded, and additional eligible candidates were recruited to meet sample size.

### Quantitative data collection

The quantitative study employed a cross-sectional online survey conducted between March and October 2024. The self-administered questionnaire collected data on demographic characteristics, diagnosis process, disease progression, treatment status, familial economic burden and mental health. Comprising 198 items, the instrument incorporated three validated scales: two assessing psychological status and one measuring quality of life. The survey was administered electronically via WenJuanXing, a web-based survey platform. Participants accessed the questionnaire through WeChat, using a dedicated link and QR code distributed by the FSHD Community Network.

Given the absence of a disease-specific HRQoL instrument for FSHD, the EuroQol five-dimension five-level (EQ-5D-5 L) was utilized as a simple, valid, and standardized measure of health status. This tool assesses five distinct dimensions: mobility, self-care, usual activities, pain/discomfort, and anxiety/depression. Each dimension is rated across five levels of severity (from 1, indicating no problems, to 5, representing extreme problems or inability to perform), generating 3,125 possible health states. The EQ-5D-5 L has been widely validated in the Chinese population and demonstrates strong psychometric properties, including high internal consistency (with Cronbach’s alpha typically exceeding 0.80) and robust construct validity in chronic and rare disease contexts [14, 15]. In this study, the Cronbach’s alpha for the EQ-5D-5 L was 0.81. Additionally, a visual analogue scale (VAS) presented in a thermometer format–ranging from 0 (worst imaginable health state) to 100 (best imaginable health state) was used to capture participants’ self-rated current HRQoL. The reliability and validity of VAS have been established in various clinical contexts, showing strong correlation with the EQ-5D index to support its convergent validity.

To assess psychological status, we employed the Zung Self-Rating Anxiety Scale (SAS) and the Self-Rating Depression Scale (SDS). Both scales consist of 20 items and are scored on a 4-point Likert scale. The Chinese versions of SAS and SDS are extensively used in clinical research and have demonstrated high reliability and validity [16]. Internal consistency in the current study was also high, with Cronbach’s alpha coefficients of 0.86 for the SAS and 0.82 for the SDS. Qualitative data collection.

In contrast to quantitative research, as a widely adopted methodological approach, qualitative research seeks to understand human experiences, behaviors, and interactions through in-depth exploration. This qualitative study employed semi-structured interviews with open-ended questions among FSHD patients. Our proposed research question: “How do individuals with FSHD perceive, interpret, and navigate the multidimensional challenges related to their diagnosis, daily management, and interaction with healthcare and social systems?“.

### Data collection

The semi-structured interview guide was initially drafted by one researcher (WJ) and subsequently reviewed by a multidisciplinary expert panel, which included three FSHD patients, three neurologists, two epidemiologists, and a pharmacoeconomics specialist. This preliminary guide was then pilot-tested with 10 FSHD patients. The pilot phase identified overly technical or ambiguous questions, which were subsequently rephrased into more accessible language. The finalized guide covered three broad thematic areas: (a) description of the disease condition, (b) the impact of FSHD on daily life, and (c) its effects on quality of life. Interviews were conducted between May and August 2024, with the sample size determined by the point of thematic saturation.

Four trained qualitative researchers (WJ, YZ, YK and LX) conducted the interviews. Prior to each session, all participants were assured of anonymity and confidentiality, and oral informed consent was obtained. The interviews followed a semi-structured format, allowing interviewers to guide the discussion while providing ample space for participants to elaborate on their experiences. Follow-up clarifications were permitted via telephone or WeChat message when necessary. Interviews were conducted via the Tencent Meeting platform (Tencent, China) with video function enabled upon participant consent. Each interview lasted between 50 and 80 min. Sessions were audio-recorded, transcribed verbatim, and checked for accuracy. At the conclusion of each interview, participants were thanked for their time and valuable contribution.

### Ethics approval

This study was reviewed and approved by the Medicine Biomedical Ethics Committee of Xi’an Jiaotong University (approval no. 2013). We registered this study in the US National Library of Medicine clinical trials database (http://clinicaltrials.gov) on July 23, 2024, under registration no. NCT06517498.

### Data analysis

Quantitative data were summarized using descriptive statistics, presented as frequencies and percentages, as well as means with standard deviations or medians where appropriate. Qualitative data were analyzed through an inductive and deductive thematic analysis with QSR NVivo V.11. Thematic analysis is one of the most widely utilized methods for analyzing qualitative data, offering a structured yet flexible framework for identifying, analyzing, and interpreting patterns of meaning within datasets. It was used to construct a conceptual model of their findings through a series of steps, including keyword and quotation selection, coding, theming, and interpretation [17]. The initial codes were refined, with recurring themes consolidated and infrequent or redundant codes merged or removed to enhance analytical coherence. First, we employed an inductive approach to identify initial codes and categories directly from the participants’ narratives, ensuring that unique experiences were captured without pre-determined bias. The remaining themes were then deductively mapped onto the Theoretical Domains Framework (TDF). In cases where a theme corresponded to multiple domains, the contextual relevance was deliberated until a consensus was reached. Representative quotations from the interview transcripts are provided in the results table to illustrate each TDF domain.

## Results

### Quantitative study

There were 263 valid questionnaires in this study, of which 226 (85.9%) were personally filled out by FSHD patients and 37 (14.1%) were filled out by their family members on their behalf. Table 1 shows that 54.0% are female, with an average age of 33 years, and 90.1% are adults; 43% have a bachelor’s degree or above; 57.4% of patients are unmarried, 35% are unemployed and 57.4% of patients have full-time caregivers; Only 8.4% of patients have sought psychological counseling or treatment for FSHD disease, 70% of patients believe that rare disease public welfare organizations such as FSHD Community Network are very helpful to them.

**Table 1:** Characteristics of the study participants

Characteristics	FSHD, n(%) (*N* = 263)
**Gender**	
Male	121 (46.0)
Female	142 (54.0)
**Age**	
0–10	8 (3.0)
11–20	27 (10.3)
21–30	64 (24.3)
31–40	101 (38.4)
41–50	41 (15.6)
51–60	18 (6.8)
≥ 61	4 (1.5)
Mean (SD)	33.08 (11.96)
Mean age, male (SD)	32.70 (12.07)
Mean age, female (SD)	33.41 (11.91)
P-value for age by gender	0.557
**Ethnic**	
Han	248 (94.3)
Others	15(5.7)
**Education**	
Preschool	2 (0.8)
Primary school	19 (7.2)
Middle school	74 (28.1)
High school	53 (20.2)
Bachelor	106 (40.3)
Masters or above	9 (3.4)
**Marital status**	
Unmarried	151 (57.4)
Married	95 (36.2)
Divorced	14 (5.3)
Lose a spouse	3 (1.1)
**Working status**	
Students	12 (4.5)
Employment, full-time work	75 (28.5)
Employment, part-time/flexible work	36 (13.7)
Unemployed	92 (35.0)
Retirement	11 (4.2)
Others	37 (14.1)
**Relationship of caregivers to patient (*n*** **= 161)**	
Parent to the patient	93 (57.8)
Partner	28 (17.4)
Other relative to the patient	12 (7.5)
Others	28 (17.4)

The departments where FSHD patients first visit were mainly neurology and orthopedics, accounting for 65.4% and 21.7% respectively. The average age of diagnosis is 23.5 years. About 1/5 of the surveyed individuals have visited more than 5 hospitals for the diagnosis of FSHD. The average time for the diagnosis of FSHD patients is as long as 9.8 years, with a misdiagnosis rate of 57%. More than half of the patients participating in this study were of FSHD type I. About 60% of patients have other FSHD patients in their family’s three generations besides themselves, and up to 23 patients have more than 5 family members with FSHD related symptoms (Table 4). The most three commonly misdiagnosed diseases include muscle weakness (20.9%), facial paralysis (11.8%) and myasthenia gravis (10.3%).

**Table 2:** Diagnosis and treatment of FSHD patients

	FSHD patient n(%) (*N* = 263)
**First visit department in the hospital**	
Neurology	172 (65.4)
Spine Surgery	6 (2.3)
Orthopedics	57 (21.7)
Other departments	8 (3.0)
Unclear	20 (7.6)
**Diagnosed department**	
Neurology	226 (85.9)
Spine Surgery	1 (0.4)
Orthopedics	8 (3.0)
Other departments	10 (3.8)
Unclear	18 (6.8)
**Diagnosed age**	
0–10	16 (6.1)
11–20	95 (36.1)
21–30	82 (31.2)
31–40	43 (16.3)
41–50	17 (6.5)
51–60	6 (2.3)
≥ 61	3 (1.1)
Mean (SD)	23.54 (11.25)
Mean age, male (SD)	35.40 (13.15)
Mean age, female (SD)	23.95 (11.61)
P-value for age by gender	0.123
Median (IQR)	21 (16–30)
Median age, male (IQR)	21 (16–30)
Median age, female (IQR)	23 (15–30)
**Number of hospitals visited to make diagnosis**	
1	49 (18.6)
2	66 (25.1)
3	71 (27.0)
4	26 (9.9)
≥ 5	51 (19.4)
**Genetic testing**	
FSHD1	143 (54.4)
FSHD2	7 (2.6)
Unsure	32 (12.2)
No genetic testing has been conducted	81 (30.8)
**Has the patient’s family undergone genetic testing**	
Yes, with the same FSHD inheritance	68 (25.9)
Yes, but the genotype is not clear	48 (18.2)
No	147 (55.9)
**Do you know about methylation detection**	
Yes and has done detection	22 (8.4)
Yes but has not done	27 (10.3)
No	214 (81.4)
**How many patients within three generations have symptoms similar to FSHD**	
0	109 (41.4)
1	56 (21.3)
2	34 (12.9)
3	29 (11.0)
4	12 (4.6)
≥ 5	23 (8.7)

The Table 4 shows the average age of onset of muscle weakness/atrophy symptoms in FSHD patients is 15.3 years, with 24.7% of patients developing symptoms before the age of 10. The decreased lower limb strength mainly happened in the age groups of 11–20 years old (39.9%) and 21–30 years old (28.1%). More than 70% of patients reported that the disease has a moderate to severe impact on their daily lives, while 62% of patients have experienced moderate to severe progression of the FSHD in the past 3 years.

Besides, falls were reported by 74.5% of FSHD patients in daily living, with 30.0% experiencing falls 1–6 times annually. Primary fall etiologies included muscle weakness (86.7%), impaired balance (70.4%), and fatigue (22.4%). Given the absence of disease-modifying therapies for FSHD, only 168 patients (64.1% of cohort) received interventions, primarily involving traditional Chinese medicine combined with structured exercise. Regarding clinical trial participation: 74 patients (28.2%) expressed willingness, 52 (19.8%) reported hesitancy, and 7 (2.7%) declined enrollment.

**Table 3:** Disease progression of FSHD patients

	FSHD patient n(%) (*N* = 263)
**Age of first onset of muscle weakness or atrophy symptoms**	
0–10	65 (24.7)
11–19	142 (54.0)
20–29	39 (14.8)
30–39	12 (4.6)
≥ 40	5 (1.9)
Mean (SD)	15.33 (7.76)
Median (IQR)	15 (11–18)
Median age, male (IQR)	16 (12–19)
Median age, female (IQR)	14 (9.5–17)
**Age at which lower limb strength deteriorates**	
1–10	23 (8.7)
11–20	105 (39.9)
21–30	74 (28.1)
31–40	45 (17.1)
41–50	11 (4.2)
≥ 50	5 (1.9)
**Have you been injured after the last fall**	
Yes, slight injury	126 (47.9)
Yes, seriously injured	10 (3.8)
No injuries from falling	127 (48.3)
**The impact of diseases on current daily life**	
No effect	4 (1.5)
slight effect	69 (26.2)
Moderate impact	87 (33.1)
Severe impact	103 (39.2)
**Muscle pain perception score**	
Mean (SD)	2.92 (2.39)
Median (IQR)	3 (1–5)

Among the 263 FSHD patients, 158 (60.1%) completed both the Self-Rating Anxiety Scale (SAS) and Self-Rating Depression Scale (SDS), with 48.8% demonstrating moderate-to-severe anxiety and 69.6% with moderate-to-severe depression (Table 4).

HRQoL was assessed in 218 adult and 25 adolescent patients using the EQ-5D instrument. Among adult participants, the EQ-5D-5L utility index demonstrated a median value of 0.671 (interquartile range [IQR]: 0.363–0.824) with a mean of 0.542, while the Visual Analog Scale (VAS) yielded a median score of 50 points (IQR: 40–70) and a mean of 51.93. The EQ-5D-Y exhibited a mean utility value of 0.658 and VAS scores with a median of 50 (IQR: 40–70) and mean of 52.28.

**Table 4:** The outcome measurements describing the (SAS), (SDS), and (EQ-5D) of the FSHD patient

	FSHD patient *n *(%)	Male *n *(%)	Female *n *(%)
**Self-Rating Anxiety Scale, SAS (Total n = 158, Male n = 66)**			
**Mean(SD)**	48.15(1.68)	46.19 (10.25)	49.56 (10.84)
No (< 0.5)	37(23.4)		
Mild (0.5–0.59)	44(27.8)		
Moderate (0.6–0.69)	38(24.1)		
Severe(≥0.7)	39(24.7)		
**Self-rating depression scale**,** SDS (Total *n*** **= 158**,** Male *n*** **= 66)**			
**Mean(SD)**	55.04(2.16)	53.47 (14.25)	56.17 (13.58)
No (< 0.5)	29(18.4)		
Mild (0.5–0.59)	19(12.0)		
Moderate (0.6–0.69)	33(20.9)		
Severe(≥0.7)	77(48.7)		
**EQ-5D-5L dimensions (**Moderate/severe**) (*n*** **= 218**,** Male *n*** **= 99)**			
Mobility	107(49.1)		
Self-care	61(27.9)		
Usual activities	92(42.2)		
Pain/discomfort	60(27.5)		
Anxiety/depression	89(40.8)		
**EQ-5D-index mean ± SD (median)**	0.561 ± 0.341(0.671)	0.618 ± 0.327(0.723)	0.514 ± 0.346(0.617)
**EQ-5D-VAS** mean ± SD (median)	53 ± 21.63(50)	53.95 ± 19.89(50)	52.82 ± 23.05(50)
**EQ-5D-Y dimensions (**Moderate/severe**) (Total *n*** **= 25**,** Male *n*** **= 12)**			
Mobility	7(28.0)		
Self-care	7(28.0)		
Usual activities	9(36.0)		
Pain/discomfort	3(12.0)		
Anxiety/depression	5(20.0)		
**EQ-5D-index mean ± SD (median)**	0.665 ± 0.262 (0.754)	0.611 ± 0.312(0.643)	0.716 ± 0.206(0.754)
**EQ-5D-VAS** mean ± SD (median)	52 ± 25.39(50)	50 ± 31.31(60)	54 ± 19.56(50)

### Qualitative study

A total of 33 patients were interviewed in this study. They were from 28 out of all 31 provinces, municipalities, and autonomous regions of China. The mean age was 28.2 years, with 19 females (57.6%). Out of 33, 20 (60.6%) were unmarried. Thirty-one (94%) have basic medical insurance, and 67% have not purchased any commercial health insurance.

Through qualitative analysis, Three main themes were identified apprehending the multifaceted burden and unmet needs of FSHD patients. These themes included: [1] disease status; [2] disease impact; [3] unmet needs. These themes are divided in sub-themes and categories. Representative quotes illustrating the categories with the respective patient number are used to demonstrate the findings (Supplemental Table 1).

#### Theme 1: Disease status

FSHD patients described a profound burden encompassing physical symptoms, inadequate treatments, unpredictable progression, diagnostic delay, and familial inheritance.

##### Symptoms

Individuals with FSHD commonly exhibit a constellation of physical symptoms, resulting in impairments in muscle function, mobility, and quality of life. The most frequently reported symptoms include upper limb weakness, scapular winging, lower limb weakness, muscle atrophy, anterior pelvic tilt, and lumbar muscle weakness.

*Commonly observed functional impairments include reduced upper limb strength*,* characterized by an inability to elevate the arms beyond the horizontal plane*,* resulting in significant difficulty with overhead lifting tasks (Pt. 1)*

##### Disease progression

The speed of disease progression in FSHD patients varies from person to person, often exacerbated by events like childbirth, infection, injury, or even rehabilitation exercises.

*The disease progresses very quickly. In 2019*,* I could still walk and run*,* but soon I began to deform while walking. Now I completely rely on a wheelchair*,* and my waist and abdominal strength are not strong when sitting*,* so I can only use straps to tie it to the wheelchair. (Pt. 16)*

##### Limited treatment options

Patients reported that the FSHD management relied on neuroprotective agents, supplements, or traditional medicine with perceived limited efficacy.

*I mainly use some neurotrophic drugs and symptomatic treatment. (Pt. 2)*

*I have sought physiotherapy for the management of scapular and thoracic region pain. (Pt. 28)*

##### Diagnosis delay

Most patients (81.8%) reported needing to visit three or more healthcare facilities to obtain a diagnosis, highlighting the significant challenges they face in achieving accurate diagnostic outcomes.

*I was advised to see the neurology department after visiting two local tertiary hospitals.*,* where the doctor suspected I might have FSHD. I underwent further examinations at the fourth hospital*,* which resulted in a high suspicion of FSHD. The diagnosis was ultimately confirmed through genetic testing at the fifth hospital (Pt. 20)*

##### Family history

A strong family history highlighted the condition’s hereditary nature and its ripple effects.

*Five people in my family have symptoms of fshd*,* three of whom were diagnosed through genetic testing (Pt. 7)*

#### Theme 2: Disease impact

FSHD has a multidimensional and profound impact on patients. It affected their life, work, education, family, finances, and mental health.

##### Daily life and social integration

FSHD severely impairs patients’ physical mobility and social integration. Fearing external scrutiny or judgment, many patients reduce their outings, gradually becoming socially isolated. Moreover, the disease imposes severe restrictions on basic daily activities, such as carrying objects, handling groceries, and even combing one’s hair.

*I’m unable to walk normally and lift things high. I’m basically disconnected from society now—I rarely go out. These days*,* I spend most of my time alone*,* doing part-time work on my phone. Going to hospital made me feel uneasy. As soon as I walked in and realized I’d have to talk to people*,* I felt scared and anxious. I was worried I wouldn’t be able to do what the doctor asked of me. (Pt. 22)*

*I experienced derision from peers*,* and differential treatment within the educational setting (Pt. 3)*

Poor sleep quality is another common issue among FSHD patients, primarily stemming from the pain and psychological stress induced by the disease. The condition also takes a significant toll on patients’ marital and interpersonal relationships.

*I have no desire to fall in love with anyone*,* no expectations for happiness or marriage. (Pt. 24)*

##### Career & educational disruption

Patients with FSHD recounted a range of challenges in maintaining their education or employment. Several shared that progressing symptoms—particularly fatigue, muscle weakness, and psychological stress—forced them to withdraw from school, resign from their jobs, or seek career changes. Some adapted by transitioning to roles with lower physical demands, while others ultimately faced early retirement or long-term unemployment.

*In March 2020*,* due to physical reasons and psychological stress*,* I resigned from work for the first time. Due to arm symptoms affecting work*,* I resigned from work for the second time in April 2024. (Pt. 26)*

*I dropped out of school from high school due to the FSHD. (Pt. 6)*

##### Family & financial strain

FSHD contributed to relationship breakdowns, conflict, discrimination from family members, and the risk of inheritance to offspring. On the other hand, although no targeted therapeutics for FSHD are currently available—thereby eliminating direct drug-related costs—patients and their families continue to face a considerable economic burden. This is primarily attributable to two factors: firstly, the cumulative expenses associated with medical care, transportation, and accommodation during the often prolonged diagnostic journey and subsequent management. Furthermore, and more significantly, it arises from the sustained loss of income due to long-term unemployment or an inability to maintain gainful employment as a consequence of the disease.

*My husband had an affair during our marriage because he couldn’t accept my disease*,* which ultimately resulted in divorce. (Pt. 11)*

*We have frequent quarrels among family members due to disease and financial pressure. (Pt. 9)*

##### Psychological stress

The FSHD patients commonly experience anxiety, insecurity, and depression. The long-term suffering caused by the illness, social misunderstanding and discrimination, as well as economic pressure, can all induce psychological issues of varying degrees in patients. Almost half of participants felt a sense of injustice or helplessness, as well guilty related to an impaired capacity to support their families.

*I am highly anxious of my appearance and facing strange looks*,* I reluctance to engage in social interactions. (Pt. 14)*

*Due to this illness*,* I experience a lot of negative emotions every day*,* I feel I am depressed. (Pt. 20)*

#### Theme 3: Unmet needs

##### Medical aspect

The paramount needs for FSHD patients lie in diagnosis and treatment. Key diagnostic challenges requiring resolution include frequent misdiagnosis, the need for repetitive testing across multiple institutions, and prolonged diagnostic timelines. Regarding treatment, there is a critical need for the development of accessible and affordable targeted drugs. Furthermore, there is a pressing demand for more equitable distribution of medical resources, enhanced clinical capabilities in underserved regions, and simplifying reimbursement procedures and increasing reimbursement rates.

*I just hope this disease doesn’t progress any further. And I hope this medicine can be released as soon as possible. I’m afraid I won’t be able to walk in a year or two. (Pt. 22)*

*I hope my leg muscles can get better*,* and that my scapular retraction improves too. (Pt. 13)*

##### Social aspect

While societal awareness of rare diseases has improved a lot in recent years, it remains insufficient. For example, the widespread lack of barrier-free infrastructure—such as dedicated access routes and accessible restrooms in public spaces. The patients hope that the infrastructure of society can be more friendly to people with disabilities. Additionally, there is a substantial need to create tailored employment opportunities that match patients’ capabilities and circumstances.

*I went to the museum*,* it was extremely challenging due to the lack of wheelchair accessibility; the only entrance involved descending a flight of stairs. I asked several people for assistance*,* but they refused*,* likely suspecting a scam. Ultimately*,* I had to sit down and carefully move myself down the stairs one step at a time. (Pt. 24)*

##### Government aspect

FSHD patients look to governmental authorities to implement policies that support drug development, accelerate clinical trials, and advance associated research for rare diseases. Concurrently, they call for the introduction of policies to safeguard the rights and interests of rare disease patients. Enhancing public education and awareness-raising initiatives is also regarded as essential to foster greater societal understanding and support.

## Discussion

This is the first representative exploratory study that provides insight into the multifaceted burden and lived experiences of Chinese patients with FSHD. By integrating quantitative assessments with qualitative inquiry, this research provides a holistic understanding of the substantial disease burden, diminished quality of life, and myriad challenges—including therapeutic barriers and unmet needs—confronted by this patient population. The findings offer critical insights that can guide future therapeutic interventions and policy development.

The disease exerts a profound impact on patients’ lives, with its progression being relatively noticeable. More than 70% of patients reported that FSHD has a moderate to severe impact on their daily lives, while 62% report experiencing moderate-to-severe disease progression within the past three years. Both quantitative and qualitative findings underscored substantial gaps in the diagnosis of FSHD. The mean diagnostic delay spans 9.8 years, accompanied by a misdiagnosis rate of 57%. Patients frequently undergo numerous clinical consultations and an extensive array of investigations before a definitive diagnosis is established. These delays contribute to postponed interventions and exacerbate the financial burden associated with the disease. Furthermore, participants reported that FSHD symptoms severely impair physical functioning—ranging from disruptions to normal sleep to limitations in self-care abilities and participation in daily activities.

Notably, FSHD exhibits a hallmark familial clustering, with approximately 60% of patients reporting other affected members within three generations of their pedigree. This hereditary pattern is further underscored by the finding that as many as 23 patients described having over five relatives manifesting FSHD-related symptoms. Among patients with typical age of onset, systemic manifestations are rare and mostly subclinical. Involvement of cardiac and respiratory muscles is similarly uncommon, highlighting the potential benefit of early and accurate diagnosis. Therefore, genetic testing —which provides definitive diagnostic confirmation—should be pursued not only in cases with classic presentation but also in those with atypical clinical features [18].

The predominant unmet need among participants was the approval of targeted therapies for FSHD—treatments that can address the condition and slow its progression. Currently, no curative interventions are available for FSHD, and disease-modifying treatments have not yet advanced beyond the clinical trials stage [19]. The present approach to manage FSHD primarily involves multidisciplinary supportive measures, focusing on physical therapy and rehabilitation exercises, optimizing pain control, and daily functioning [8]. In cases of severe pathology, physical therapy, as well as assistive devices, are useful and can be tailored to the specific needs of the patient [20, 21]. Patients might benefit from the use of orthopaedic interventions, such as scapular fixation surgery, leg braces for foot drop and corsets for back support [20, 22]. Exercise has shown an important impact in relieving symptoms and proper management may delay progression [23–25]. Hence, patients with FSHD should be encouraged to engage in low-intensity aerobic exercise, which has a beneficial effect on chronic fatigue, physical activity and fitness.

A significant proportion of FSHD patients endorsed symptoms consistent with moderate-to-severe anxiety (approximately 50%) and depression (69.6%). These quantitative findings are substantiated by qualitative data, wherein patients frequently described profound feelings of helplessness, emotional distress, and consequent social withdrawal. Importantly, patient narratives also revealed the development of adaptive coping mechanisms and emotional resilience, suggesting potential therapeutic targets for integrated psychosocial support. This aligns with other studies that emphasize the need to address emotional factors in muscle diseases, as well as those that have identified a link between emotional distress and perceptions of disease severity [26, 27]. The significant emotional burden in FSHD patients warrants greater clinical focus, particularly as it constitutes a tractable component of the disease experience. Targeted interventions—spanning psychosocial support, education, and evidence-based mental health treatments—offer a viable pathway to mitigate this aspect of suffering and improve overall patient outcomes [26, 28].

Regarding insurance coverage and social safeguards, existing mechanisms, including certain insurance schemes and subsidies, prove inadequate in addressing the comprehensive needs of FSHD patients. In addition, numerous patients have purchased insurance policies only to subsequently discover that FSHD-related expenses are excluded from coverage. Within the Chinese context, considering the nation’s current socioeconomic development stage and the affordability constraints for all stakeholders, it is recommended to adopt an integrated support model. This model should converge medical security systems, social assistance programs, and charitable aid to foster the development of viable commercial health insurance products specifically designed for rare diseases like FSHD [29]. FSHD patients have high expectations for robust reimbursement support for potential targeted medications. Feasible multi-payer models, including local “special rare disease funds,” critical illness insurance, inclusive commercial health insurance, and government-led medical assistance programs, are currently being piloted or implemented to establish a sustainable coverage framework for patients with rare diseases [30].

Consistent with prior research, data derived from the EQ-5D instrument demonstrated marked impairments in the domains of self-care, mobility, usual activities, and pain/discomfort—a finding substantiated and richly detailed by subsequent patient interviews. These results collectively underscore the severe and multidimensional deterioration in health-related quality of life associated with FSHD. In light of this significant burden, a clear demand emerges from patients for enhanced supportive care, including improved access to assistive devices and the implementation of tailored, sustained psychological intervention programs. Furthermore, effective management of core symptoms such as chronic pain and fatigue is essential, given their substantial exacerbating effect on psychological distress [31, 32].

Financial burden emerged as one of the most critical personal challenges due to medical expenditures. And FSHD significantly impairs work capacity, leading to reduced efficiency, job loss, and an inability to sustain employment. Qualitatively, this occupational decline is attributed to a multifactorial interplay of workplace discrimination, progressive physical limitations, and emotional exhaustion.

To address the challenges identified in this study, we propose the following integrated recommendations: (1) Implement standardized diagnostic pathways to ensure timely and accurate FSHD diagnosis, thereby reducing diagnostic delays and misdiagnosis. (2) Facilitate patient access to international multi-center clinical trials to accelerate novel therapy development and approval in China, while promoting rehabilitation to enhance quality of life. (3) Establish transparent, inclusive insurance frameworks for rare diseases, complemented by centralized information platforms to ensure coverage clarity and prevent misinformation. (4) Systematically integrate psychosocial support services—including psychological interventions, counseling, and peer support—into standard FSHD care. (5) Launch awareness campaigns to foster an inclusive social environment, create adaptable employment opportunities, and mitigate stigma, thereby improving social integration for individuals with rare diseases. Future research should prioritize longitudinal and intervention-based studies to deepen our understanding of FSHD and ultimately promote clinical practice.

### Strengths and limitations

To our knowledge, this is the first study to apply a convergent parallel mixed-methods design in assessing the multidimensional burden of FSHD in China. Nationwide recruitment through a major patient advocacy organization enhanced geographic diversity and sample representativeness, while prospective registration strengthened the transparency and rigor of the research process. However, despite these strengths, several limitations warrant consideration. Reliance on a single patient network for self-reported data—even with a geographically stratified sampling strategy—introduces potential selection and self-selection biases, which constrain the generalizability of the findings. Moreover, although data saturation was achieved, the specific characteristics of the sampled participants may have limited the capture of the full spectrum of issues relevant to the broader FSHD population. The cross-sectional design further precludes causal inferences regarding the observed associations. Finally, the absence of a validated FSHD-specific instrument means that the patient-reported outcomes employed may not fully capture the disease’s distinctive impact on health-related quality of life.

## Conclusion

This mixed-methods investigation elucidates the multifaceted burden experienced by patients with FSHD, encompassing significant disease-related impairments, psychological impacts and critical delays in diagnosis. Key challenges and unmet needs identified include the absence of targeted therapies, inadequate insurance coverage, insufficient psychological support, and work capacity decline. To address these gaps, we recommend a concerted effort to: enhance diagnostic efficiency; accelerate the research and development of targeted therapies; establish comprehensive and inclusive insurance frameworks for rare diseases; integrate mental health services into medical care; and foster social inclusion alongside workplace accommodations, with the ultimate goal of improving patients’ quality of life. Further longitudinal and intervention studies are essential.

## Supplementary Information

Below is the link to the electronic supplementary material.

Supplementary Material 1

## Data Availability

The data that support the findings of this study are available from the corresponding author upon reasonable request.

## Abbreviations

FSHD: Facioscapulohumeral muscular dystrophy
CORD: Chinese Organization for Rare Disorders
