# Supplementary material for: Assessing the multidimensional burden of facioscapulohumeral muscular dystrophy through patient-reported outcomes and experience
Source: J Patient Rep Outcomes. 2026 Feb 25;10:50. doi: 10.1186/s41687-026-01026-z (PMC13038818; doi:10.1186/s41687-026-01026-z)
Supplement: Supplementary file 1 — Supplementary Material 1 [file 41687_2026_1026_MOESM1_ESM.docx]

Supplemental Table1 Representative Quotations from Patient-Reported Burden and Needs in FSHD

| Theme | Sub-themes | Example Quotations |
| --- | --- | --- |
| **Disease Burden** | Symptoms | “Both lower limbs have difficulty in climbing stairs, walking is unstable, and it's necessary to hold onto the handrail.” |
|  |  | “The protrusion of the scapula is obvious.” |
|  |  | “Had poor abdominal and back strength before, and it was hard to do sit-ups.” |
|  |  | “Symptoms of pectus excavatum appeared after 2022.” |
|  | Disease Progression | "When I was 13 to 15 years old, I could still run. After 16 years old, I couldn't walk anymore. At 18 years old, I had difficulty climbing stairs. After 20 years old, I had difficulty walking. After 22 years old, I basically didn't go out and stayed at home all the time. At 23 years old, my waist started to hurt. At 25 years old, I started using a wheelchair, but I can still walk a little when I go out. Now I rely entirely on a wheelchair. "(Pt. 9) ." |
|  |  | "After giving birth my kid in 2017, the abdominal muscles were damaged, climbing stairs became more difficult, and standing up after squatting became more challenging." |
|  |  | "After contracting COVID-19 in 2022, the strength of the left leg worsened and I’m unable to climb stairs." |
|  |  | “After an excessive rehabilitation exercise, the symptoms worsen significantly.”  “At the age of 18, I sustained a fall from a balcony, resulting in an arm injury. Following suboptimal recovery, a constellation of symptoms emerged progressively, with greater prominence in the upper limbs.” |
|  | Limited Treatment Options | “After confirmation, I go to the hospital for a review once a year and receive some nerve-nutrient drugs.”  “I have taken some vitamin supplements.” |
|  |  | “I’ve tried acupuncture and moxibustion treatments, and taken traditional Chinese medicine. For me, I’ve relied on Chinese medicine and nourishing remedies to improve and boost my health, buy the tangible, substantive improvements for FSHD remain limited.” |
|  |  | “I use a girdle for abdominal protrusion, improving quality of life and appearance through physical support.” |
|  |  | “After confirmation in August 2022, I had a pectoralis major muscle transfer surgery to improve the symptoms of inability to lift the right arm and protruding scapula.” |
|  |  | “I started aerobic exercise in 2017, which had some effect on improving symptoms. Previously I needed to wear a waist support and foot orthotics when going out, now I basically do not need any support, and I do not feel particularly tired after walking for a long time.” |
|  | Diagnosis Delay | "Initially I went to the orthopedic department but was not diagnosed. Then I visited a neurology department in Beijing and had an electromyogram and muscle biopsy. After that, I was diagnosed with FSHD." |
|  |  | " I was diagnosed with FSHD until the antenatal screening" |
|  |  | In 2019, I was initially misdiagnosed with myasthenia gravis. Since genetic testing did not yield conclusive results, I later went to another Hospital, where I received the second misdiagnosis of Duchenne muscular dystrophy (DMD). Ultimately, I went to Beijing, where the diagnosis of FSHD was confirmed through genetic testing and a muscle biopsy. |
|  | Family History | "My mother has passed away, but I remember she was always in a wheelchair and had some similar symptoms." |
|  |  | “My mother exhibits symptoms of FSHD. Among my siblings, only my younger sister is unaffected. My older sister is wheelchair-bound, with more severe symptoms, and two of her five children have been diagnosed with FSHD. My older brother, who had extremely severe symptoms, passed away—from what I can recall, he never walked independently. My second aunt can only walk in a squatting posture. Her eldest daughter has intellectual impairments, while her second daughter also walks in a squatting position due to complete muscle atrophy. Her third daughter developed walking posture abnormalities in the past two years. Her eldest son shows obvious facial muscle symptoms, and her youngest son walks on tiptoes with some facial manifestations as well. My youngest aunt has one son and one daughter, both of whom are currently symptom-free." |
| **Disease Impact** | Daily life and social integration | "The symptoms have a great impact on life. Now I’m unable to walk normally and lift things high. I’m quite thin, and need care in many aspects." |
|  |  | "FSHD has significantly compromised their daily life and work capacity.” |
|  |  | “My upper limbs can reach overhead, but simple tasks like picking up objects, carrying groceries, combing my hair, tying a ponytail, or blow-drying my hair feel strenuous. When I lift things with my left hand, it can only stay in a vertical position and won’t bend backward at all” |
|  |  | "I have no plan to get married or have children in the future." |
|  |  | "The disease has a certain impact on sleep. Over time there is a feeling of oppression and pain in the shoulders and back. I need to get up and move around before being able to sleep again."  "The sleep quality is quite poor, mainly affected by psychological stress." |
|  |  | "The shoulder muscles are weak and unable to lift, I was criticized by teachers and mocked by classmates and suffers discrimination." |
|  |  | "Following a prior pregnancy that resulted in an elective termination, a successful live birth was ultimately achieved through in vitro fertilization (IVF). But I have experiencing significant hardship in the pursuit of parenthood, primarily due to concerns heritability. " |
|  | Career & educational disruption | In March 2020, due to physical reasons and psychological stress, I resigned from work for the first time. Due to arm symptoms affecting work, I resigned from work for the second time in April 2024." |
|  |  | "Due to direct physical condition and psychological stress, I dropped out of school in junior high." |
|  | Family & Financial Strain | "My family is burdened with liabilities estimated at 200,000 Chinese Yuan." |
|  |  | "There are many confirmed cases in the family, and this has put pressure on us, leading to frequent quarrels among family members." |
|  |  | "I face discriminatory attitudes and behaviors from my mother-in-law and husband. " |
|  |  | "The FSHD has a great impact on the family. I worried about the disease inheritance, and also worried about the worsening of symptoms for myself and my family." |
|  |  | “I went to Beijing to access medical care. The cumulative costs were substantial, encompassing thousands of RMB for transportation, tens of thousands for accommodation and sustenance, and additional significant fees for intermediary services to secure appointments. The surgical procedure itself cost nearly 100,000 RMB, resulting in a total financial burden described as overwhelming.” |
|  | Psychological stress | "FSHD has profoundly impacted my psychological state. While intense negative emotions were predominant initially, they have subsided and now occur only intermittently. However, the inability to complete certain tasks still triggers mood deterioration, impaired concentration, and significant distress." |
|  |  | “The insidious progression of the disease brings about a deep sense of helplessness, stemming from the experience of witnessing my own transformation from a healthy individual to a patient.” |
|  |  | "I exhibits significant preoccupation with my appearance, which contributes to feelings of inferiority." |
|  |  | "Every aspect of the my mood is governed by the disease. Chronic adversity has resulted in severe introversion and a perpetually furrowed brow." |
|  |  | “I experience profound guilt towards my family, stemming from the long-term inability to secure employment due to my condition. This has prevented me from providing tangible support to my parents, which reinforces a pervasive sense of being useless and without value.” |
| **Unmet Needs** | Medical aspect | "Repeated examinations in different hospitals led to high costs and great suffering. Hope the disease could be diagnosed timely." |
|  |  | "I hope there can be some assistive devices to help me stand up again and achieve independent living." |
|  |  | "The disease has a significant impact on physical appearance, such as walking posture and abdominal protrusion. I really hope to improve these symptoms." |
|  |  | "Assuming new drugs are developed, it is hoped that they will not be prohibitively expensive." |
|  |  | "As there is no definitive treatment currently available, hope to have affordable and accessible supportive and centered on rehabilitation." |
|  |  | "I hope the reimbursement ratio can be increased”. |
|  |  | “My biggest need is to restore the strength in my legs so that I can take care of my basic daily needs.” |
|  |  | "If any way to help dealing with my own psychological struggles, I believe that addressing psychological stress would do more to improve my state of being than any treatment plan." |
|  |  | "Regional disparities in medical resources need to be improved. Medical resources in remote areas are relatively backward, and it is inconvenient for medical treatment and follow-up visits." |
|  |  | “I purchased a commercial insurance plan (specifically, not a critical illness insurance) in 2016. However, it does not cover any claims related to FSHD. So I hope the insurance can cover more reimbursement content and a higher reimbursement ratio.” |
|  | Social Aspect | "I hope society could introduce suitable work opportunities for patients with rare diseases, and protect our basic rights." |
|  |  | “I encountered significant barriers in air travel, from boarding aircraft to using airport shuttle buses. Furthermore, public venues like museums often lack adequate facilities. On one visit, I had to climb four floors to find a restroom, only to discover that the sole accessible stall was out of service for maintenance. |
|  |  | “I hope that the infrastructure of society can be more friendly to people with disabilities.” |
|  |  | "There are few channels to obtain disease-related information, and the information understood is limited." |
|  |  | "Boarding an airplane and taking a shuttle bus is very inconvenient for people with disabilities. At the museum, I have to walk up four floors to find a restroom, but there was no disabled-accessible one, and the only one available was under maintenance." |
|  | Government Aspect | "It is recommended that the government formulate supportive policies to incentivize drug development for rare diseases. Without such national-level intervention, individual efforts will remain insufficient." |
|  |  | “I hope the government could actively facilitate the accelerated introduction of new therapies and the advancement of clinical trials.” |
|  |  | “It is vital to enhance public education on rare diseases to cultivate greater societal understanding and support.” |
